# Boosting of Redox-Active Polyimide Porous Organic Polymers with Multi-Walled Carbon Nanotubes towards Pseudocapacitive Energy Storage

**DOI:** 10.3390/nano14171388

**Published:** 2024-08-26

**Authors:** Tian Zhou, Yu Yuan, Luyi Xiao, Wei Ding, Yong Wang, Li-Ping Lv

**Affiliations:** 1 School of Environmental and Chemical Engineering, Shanghai University, 99 Shangda Road, Shanghai 200444, China; 2 Key Laboratory of Organic Compound Pollution Control Engineering (MOE), Shanghai University, 99 Shangda Road, Shanghai 200444, China

**Keywords:** porous organic polymers, redox-active polymers, polyimide, pseudocapacitance, multi-walled carbon nanotubes

## Abstract

Redox-active porous organic polymers (POPs) demonstrate significant potential in supercapacitors. However, their intrinsic low electrical conductivity and stacking tendencies often lead to low utilization rates of redox-active sites within their structural units. Herein, polyimide POPs (donated as PMTA) are synthesized in situ on multi-walled carbon nanotubes (MWCNTs) from tetramino-benzoquinone (TABQ) and 1,4,5,8-naphthalene tetracarboxylic dianhydride (PMDA) monomers. The strong π–π stacking interactions drive the PMTA POPs and the MWCNTs together to form a PMTA/MWCNT composite. With the assistance of MWCNTs, the stacking issue and low conductivity of PMTA POPs are well addressed, leading to the obvious activation and enhanced utilization of the redox-active groups in the PMTA POPs. PMTA/MWCNT then achieves a high capacitance of 375.2 F g^−1^ at 1 A g^−1^ as compared to the pristine PMTA POPs (5.7 F g^−1^) and excellent cycling stability of 89.7% after 8000 cycles at 5 A g^−1^. Cyclic voltammetry (CV) and in situ Fourier-Transform Infrared (FT-IR) results reveal that the electrode reactions involve the reversible structural evolution of carbonyl groups, which are activated to provide rich pseudocapacitance. Asymmetric supercapacitors (ASCs) assembled with PMTA/MWCNTs and activated carbon (AC) offer a high energy density of 15.4 Wh kg^−1^ at 980.4 W kg^−1^ and maintain a capacitance retention of 125% after 10,000 cycles at 5 A g^−1^, indicating their good potential for practical applications.

## 1. Introduction

Due to their high power density, fast charge/discharge rate, excellent safety, and long-term cycling stability, supercapacitors (SCs) have been widely studied and used independently or as supplement power supply to the currently star energy storage system, i.e., lithium ion batteries (LIBs) [1]. However, their relatively low energy density as compared to LIBs remains a critical issue limiting their practical application [2]. In recent decades, there has been tremendous progress in investigating and understanding SCs with high energy densities, and in such work, developing new electrode materials that can provide abundant pseudocapacitance is regarded as one of the most effective strategies. Indeed, the utilization or introduction of electroactive molecules to acquire abundant pseudocapacitance represents an efficient way to achieve high-performance SCs. Different from the electric double-layer capacitors (EDLCs) based on physical charge storage theory, the pseudocapacitive energy storage process is based on a fast Faraday reaction which can provide higher energy storage capacity than EDLCs and thus SC devices with high energy density can be obtained [3,4,5,6].

One strategy to achieve this goal is the utilization of pseudocapacitive materials that can take advantage of reversible surface or near-surface Faradaic reactions to store charges [7]. This allows them to surpass the capacity limitations of electrical double-layer capacitors and the mass transfer limitations which are often involved in batteries. Porous organic polymers (POPs) represent one class of promising pseudocapacitive materials with great potential to be well designed [8]. Covalent organic frameworks (COFs), for example, as a kind of popular POPs, have advantages such as adjustable structure, abundant pseudocapacitive active sites, high porosity, and ordered inner spaces that facilitate ion diffusion, making them good candidates as electrode materials [9,10,11,12]. However, the inherent electrochemical inertness of POP-based materials, namely, their poor conductivity, has become the key factor to restricting their use as electrode materials. In addition, POP materials are often easily accumulated due to interlayer interactions, such as π–π stacking interaction and other intermolecular forces, which causes energy storage active sites to be buried deeply and cannot play a sufficient role in energy storage characteristics. A special structural configuration or the modulation of interlayer interactions to expose more active sites to enhance the supercapacitor performance are therefore highly demanded [13,14,15]. In addition, it is also very important to find a solution that can simultaneously solve the problems of poor conductivity and stacking of POP materials. Among these, the strategy of compositing with carbon materials is most commonly used to improve the conductivity of electrode active materials and meanwhile help alleviate the stacking problem, thus providing a favorable way to prepare supercapacitor devices with high energy density [16,17]. Nevertheless, the utilization of carbon materials to magnify the performance of POP-based electrodes still requires careful material design with the boosting mechanism demonstrated in detail to achieve optimal performance. In addition, the study on the effect of interfacial interaction between the active substances and the carbon materials on their efficiency as electrode materials for supercapacitors is also of great importance and requires focused investigation [18].

Herein, pseudocapacitive polyimide POPs rich in quinone functional groups are in situ-grown on MWCNTs. The in situ synthesis of POPs on MWCNTs can effectively alleviate their stacking problem, and meanwhile makes the POPs and MWCNTs closely bonded to achieve an enhanced conductivity with improved electron transfer efficiency. The POP/MWCNT composite then achieves boosted electrode performances as compared to the pristine POPs. A detailed study on electrode reaction kinetics together with in situ FTIR technology are conducted to demonstrate the energy storage process as well as the boosting mechanism of the POP/MWCNT electrode with high specific capacitance and long cycle stability.

## 2. Materials and Methods

Materials. 1,2,4,5-benzenetetracarboxylic anhydride (PMDA, 99%) and 1,3,5-trimethylbenzene (C_9_H_12_, 98%) were purchased from Shanghai Aladdin Biochemical Technology Co., Ltd., Shanghai, China. Carboxyl-functionalized multi-walled carbon nanotubes (COOH-MWCNTs, 99%) and acetylene black (C, battery grade) were obtained from Jiangsu XFNANO Materials Tech Co., Ltd., Nanjing, China. 2,3,5,6-tetraaminobenzoquinone (TABQ, 98%) was purchased from Jilin Chinese Academy of Sciences—Yanshen Technology Co., Ltd., Changchun, China. Activated carbon (AC, YP-50F) was obtained from Kuraray Co., Ltd., Tokyo, Japan. 1,4-dioxane (C_4_H_8_O_2_, analytical purity), glacial acetic acid (C_2_H_4_O_2_, 37%), and sulfuric acid (H_2_SO_4_, 95–98%) were provided by Sinopharm Chemical Reagent Co., Ltd., Shanghai, China.

Synthesis of PMTA POPs and PMTA/MWCNTs. The synthesis of PMTA POPs and PMTA/MWCNTs was carried out according to the reported literature [9]. In a typical procedure, 11.8 mg of TABQ, 30.5 mg of PMDA, and a certain amount of MWCNTs were placed in a 25 mL of hydrothermal kettle, followed by the addition of 1.5 mL of 1,4-dioxane and 0.5 mL of 1,3,5-trimethylbenzene. The obtained mixture was then ultrasonicated for 30 min, followed by the addition of 0.2 mL of acetic acid (3 M), and the reaction was carried out in a vacuum oven at 120 °C for 3 days. After reaction, the hydrothermal kettle was cooled to room temperature and the black product was collected and washed three times thoroughly with acetone and deionized water. After drying at 60 °C for 24 h, the final product (PMTA/MWCNTs) was obtained. The synthetic process of PMTA was identical to the synthesis of PMTA/MWCNTs except that MWCNTs were not added during the reaction process. It should be noted that the feeding mass of TABQ and PMDA was kept constant for both samples, and only the feeding mass of MWCNTs was changed to obtain PMTA/MWCNT-1, PMTA/MWCNT-2, and PMTA/MWCNT-3 with a feeding mass ratio of the total mass of the POP monomers to MWCNTs of 2:1, 4:1, and 7:1, respectively.

Material characterization. The microstructure and morphology of PMTA and PMTA/MWCNT electrode materials was observed using a JSM-7500F scanning electron microscope (SEM) and a Hitachi 7800 transmission electron microscope (TEM). The samples were dried on silicon substrates for SEM tests and coated on copper grids for TEM measurements. Energy-dispersive X-ray spectroscopy (EDS) spectra were recorded on the X-ray photoelectron spectrometer (INCAx-act) of a Hitachi SU1510 SEM. The elemental composition and valence state of the electrode materials were characterized using an X-ray photoelectron spectrometer (XPS, Thermo Fisher Nexsa, Waltham, MA, USA). The XPS spectra were recorded in the normal photoemission registration mode at hν = 1486.6 eV with an energy resolution of 0.5 eV. All X-ray photoelectron spectra were fitted by the Gaussian/Lorentzian convolution functions with a simultaneous optimization of the background parameters using Thermo Avantage v5.9931 software. The specific surface area and pore size distribution of the materials were determined using an automatic specific surface area analyzer (Micromeritics, ASAP 2020 Plus) and calculated based on BET (Brunauer, Emmet, and Teller) model. The thermal stability of the electrode materials was characterized using a thermogravimetric analyzer (TGA, NETZSCH, TG209 F3) in the range of 25–800 °C (N_2_), with a heating rate of 5 °C min^−1^, with samples fully dried before tests. Fourier-Transform Infrared (FT-IR) was conducted on a Nicolet iS50 instrument with all the samples dried before tests. For the in situ FT-IR tests, a custom-made electrochemical cell connecting to a CHI760E electrochemical working station was equipped with the FT-IR instrument. The FT-IR spectra were then collected in real time with an electrochemical charge/discharge process using an Attenuated Total Reflectance (ATR) mode. The X-ray powder diffraction (XRPD) patterns were recorded on a Smartlab 9 kw Rigaku X-ray diffractometer with Cu Kα radiation (λ = 0.15406 nm).

Electrochemical analysis. The electrochemical performances were evaluated in a traditional three-electrode system (working electrode: PMTA or PMTA/MWCNTs as active materials; counter electrode: platinum; reference electrode: Hg/Hg_2_SO_4_; electrolyte: 1 M H_2_SO_4_). The voltage window for the cyclic voltammetry (CV) and the galvanostatic charge–discharge (GCD) test was −0.4–0.2V, and electrochemical impedance spectroscopy (EIS) was conducted in the range of 0.01 Hz to 1000 kHz. 

The charge storage capacity of the electrode material was evaluated according to the GCD test. The specific capacitance (C), energy density (E) and power density (P) of the electrode material were calculated according to Equations (1)–(3) [19].

(1) $$C=\frac{I\cdot \Delta t}{m\cdot \Delta V},$$

(2) $$E=\frac{1}{2}\cdot C\cdot \Delta V^{2},$$

(3) $$P=\frac{E}{\Delta t}\;,$$

where C (F g^−1^) represents the specific capacitance, I (A g^−1^) represents the current density, Δt (s) represents the discharge time, ΔV (V) represents the voltage window, and m (g) represents the mass of the active material. E (Wh kg^−1^) represents the energy density, and P (W kg^−1^) represents the power density.

When assembling the supercapacitor, the mass ratio of the active material loaded on the cathode and anode was calculated according to Equation (4).

(4) $$\frac{M_{-}}{M_{+}}=\frac{C_{+}V_{+}}{C_{-}V_{-}}$$

where M_+_, M_−_, C_+_, C_−_, V_+_, and V_−_, respectively, represent the mass, specific capacitance, and voltage window of the positive and negative electrodes of the device.

To assemble the PMTA/MWCNT//AC asymmetric supercapacitors, 1M H_2_SO_4_ aqueous solution, the as-prepared PMTA/MWCNTs, commercial AC, and filter paper were used as the electrolyte, the cathode material, the anode material, and the separator, respectively.

## 3. Results and Discussions

**Figure 1:**
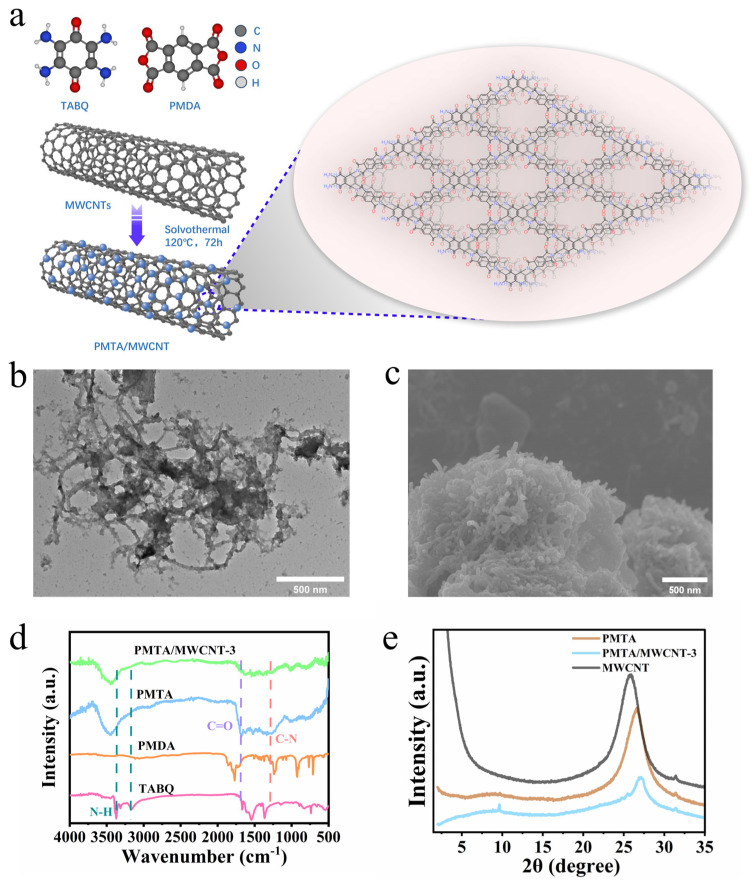
(**a**) Schematic diagram of the synthesis of PMTA POPs and the PMTA/MWCMT composite; (**b**) TEM image of PMTA/MWCNT-3; (**c**) SEM image of PMTA/MWCNT-3; (**d**) FT-IR spectra of the monomer TABQ, PMDA, the synthesized PMTA POPs, and the PMTA/MWCNT-3 composite; (**e**) XRD patterns of PMTA, MWCNTs, and the PMTA/MWCNT-3 composite.

Figure 1a indicates the preparation process of the PMTA POPs and PMTA/MWCNT composite. Both the pristine PMTA and PMTA/MWCNT composite materials were prepared using a simple solvothermal method. During the synthesis of PMTA, the reaction monomer TABQ and PMDA were first dissolved in a mixture solvent of 1,4-dioxane and 1,3,5-trimethylbenzene. Then, the two monomers reacted with each other, driven by the charge transfer interactions between the amino donor moieties from TABQ and the dianhydride acceptor moieties from PMDA, forming polyamic acid (PAA) intermediates. Thereafter, upon the addition of acetic acid as catalyst, PAA was further converted to the corresponding polyimide PMTA via intermolecular cyclization at 120 °C [20,21]. Different from PMTA POPs, the PMTA/MWCNT composite was synthesized through an in situ growth method in the presence of MWCNTs. As compared to the pristine PMTA POPs with a bulky morphology (Figure S1a,b), PMTA/MWCNTs exhibit looser structure with POPs located on the surfaces of MWCNTs (Figure 1b,c). The EDS spectrum shows the presence of C, N, and O elements, and EDS mapping further indicates their good distribution in the PMTA/MWCNT composite (Figure S2). The π–π interactions arising from the benzene rings of the monomers and the delocalized π-bonds from MWCNTs are the driving force for the nuclei growth of POPs on the MWCNT surfaces. 

In addition, the hydrogen bonding interactions arising from the functional groups, i.e., the amino and carbonyl groups in the TABQ and PMDA monomers and the carboxyl groups on the MWCNTs, can further help the in situ polymerization of the two monomers on the MWCNTs to generate the PMTA/MWCNT composite. As shown in FT-IR spectra (Figure 1d), the vibration absorption of -NH_2_ in TABQ centered at ~3377 cm^−1^ and 3172 cm^−1^ and the aromatic C-O-C in PMDA located at ~1116 cm^−1^ disappear, while the absorption peaks of C-N bonds (~1337 cm^−1^) and C=O bonds (~1689 cm^−1^) assigned to the polyimide structure appear, indicating the acylated reactions to form polyimide-based PMTA POPs [22]. When composting with MWCNTs, PMTA/MWCNTs exhibit similar characteristic peaks to the pristine PMTA, indicating negligible influence of the MWCNTs on the reactions to form PMTA POPs. The XRD patterns (Figure 1e) display broad peaks at around ~25–30° for PMTA which overlap with (002) planes of MWCNTs and can be attributed to the π−π staking between interlayers of PMTA, indicating its low crystallinity [23,24].

**Figure 2:**
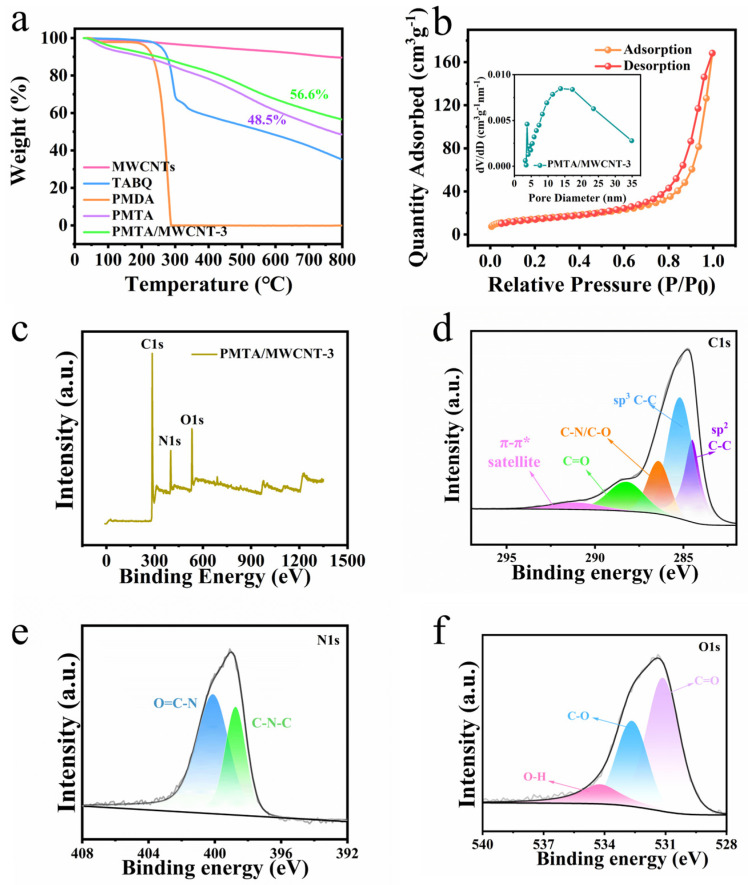
(**a**) TG analysis of MWCNTs, TABQ, PMDA, PMTA, and PMTA/MWCNT-3; (**b**) Nitrogen adsorption/desorption isotherms of PMTA/MWCNT-3; (**c**–**f**) XPS spectra of PMTA/MWCNT-3 material: (**c**) Survey; (**d**) C 1s; (**e**) N 1s; (**f**) O 1s.

Figure 2a indicates the thermal stability of PMTA POPs and PMTA/MWCNTs. The small decrease in weight from room temperature to 100 °C is due to the loss of residual solvent or adsorbed water for the samples. As temperature increases to 300 °C, the monomer TABQ and PMDA start to degrade with obvious mass loss observed. In contrast, PMTA and PMTA/MWCNTs exhibit a gentle mass loss up to 800 °C, further indicating the successful polymerization of the monomers and the improved thermal stability of the PMTA POPs with the addition of MWCNTs. Based on the nitrogen adsorption–desorption isotherms shown in Figure 2b and Figure S3, the BET specific surface area of PMTA and PMTA/MWCNTs are calculated to be ~16.4 and ~51 m^2^ g^−1^, respectively, indicating that the presence of MWCNTs helps alleviate the stacking issue of PMTA to expose more surfaces. In addition, the pore size distribution was observed to center at round 6–20 nm for PMTA which is similar to that of the PMTA/MWCNT composite, i.e., ~3.8 nm and ~13.7 nm, implying mesopores existing in both samples. These mesoporous structures are conducive to provide favorable contact to electrolytes and paths for the transportation of electrolyte ions [25]. The elemental composition and valence states of PMTA/MWCNT composites were further analyzed using XPS spectroscopy. The XPS survey spectra show characteristic peaks of C, N, and O (Figure 2c). In particular, C=C (~284.5 eV), C-C (~285.2 eV), C-N/C-O (~286.4 eV), and C=O (~288.2 eV) are observed in the C 1s spectrum (Figure 2d) [26,27,28,29,30]. A small π-π* satellite peak at around ~291 eV can be corresponding to the large conjugated system and sp2-character in MWCNTs [22,27]. The N 1s spectrum displays two main peaks located ~398.8 eV and 400.2 eV, corresponding to C-N-C and N-C=O, respectively (Figure 2e) [31]. The binding energy of ~531.1 eV, 532.6 eV, and 534.2 eV in the O 1s spectrum corresponds to C=O, C-O, and O-H, respectively (Figure 2f) [31,32]. All the aforementioned analyses indicate the successful synthesis of PMTA which grew in situ on MWCNTs. With the assistance of MWCNTs, the electron transport in PMTA is expected to accelerate with the redox-active groups activated, achieving a capacitance enhancement. Moreover, the aggregation and stacking of PMTA can also be well alleviated with the help of MWCNTs, thus leading to a higher exposure of active sites in the POPs. 

**Figure 3:**
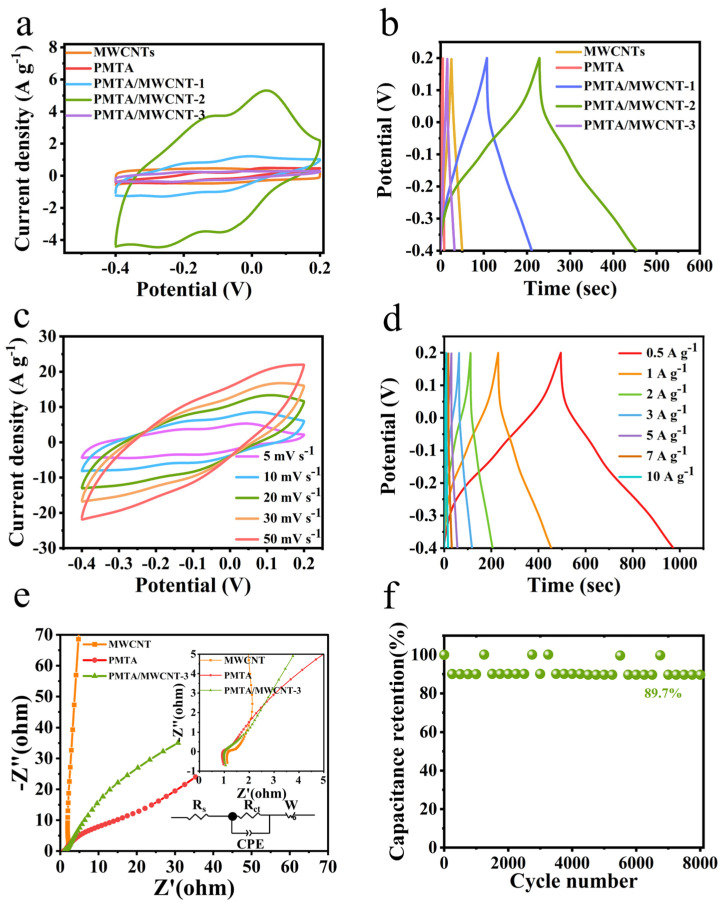
(**a**) CV curves and (**b**) GCD curves of MWCNTs, PMTA, PMTA/MWCNT-1, PMTA/MWCNT-2, and PMTA/MWCNT-3 at a scan rate of 10 mV s^−1^ and a current density of 1 A g^−1^, respectively; (**c**) CV curves of PMTA/MWCNT-3 at 5, 10, 20, 30, and 50 mV s^−1^; (**d**) GCD curves of PMTA/MWCNT-3 at 0.5, 1, 2, 3, 5, 7, and 10 A g^−1^; (**e**) EIS plots of MWCNTs, PMTA, and PMTA/MWCNT-3 (the illustration shows the high frequency area); (**f**) cycling stability of PMTA/MWCNT-3 at 5 A g^−1^.

The electrochemical performances of MWCNTs, PMTA, and PMTA/MWCNT composites were first investigated in a typical three-electrode system with 1M H_2_SO_4_ electrolyte. Figure 3a shows the CV curves of MWCNTs, PMTA, PMTA/MWCNT-1, PMTA/MWCNT-2, and PMTA/MWCNT-3 at a scan rate of 10 mV s^−1^. It is clear to observe that PMTA/MWCNT-3 exhibits the largest CV integral area, indicating its highest specific capacitance compared to other electrode samples. Meanwhile, the CV curve of PMTA/MWCNT-3 is approximately rectangular in shape and exhibits two pairs of broad redox peaks, demonstrating the co-existence of double-layer capacitive and pseudocapacitive energy storage mechanisms. The appeared redox peaks between −0.3 V and 0.3 V can correspond to the electrochemical reactions of carbonyl groups in PMTA/MWCNTs [23,33,34]. Based on the GCD profiles collected at 1 A g^−1^ shown in Figure 3b, the specific capacitances of MWCNTs, PMTA, PMTA/MWCNT-1, PMTA/MWCNT-2, and PMTA/MWCNT-3 are calculated to be 11.8, 5.7, 41.3, 172.3, and 375.2 F g^−1^, respectively. These observations indicate the boosted energy storage capability of PMTA with the assistance of MWCNTs. In addition, an appropriate mass ratio of PMTA to MWCNTs is significant to achieve the improved capacitance of the electrode. Figure 3c and Figure S4a,c show the CV curves of PMTA/MWCNT composites at various scan rates of 5~50 mV s^−1^ with multiple pairs of redox peaks for all three electrodes, implying their pseudocapacitive property. Figure 3d and Figure S4b,d further show their GCD profiles at different current densities between 0.5 and 10 A g^−1^. All the shapes of the GCD curves are approximately symmetrical triangles, indicating good charge–discharge reversibility for the PMTA/MWCNT electrode materials [35]. Based on the GCD curves, the specific capacitance of PMTA/MWCNT-3 is calculated to be ~398 F g^−1^ at 0.5 A g^−1^ and still remains at 116.7 F g^−1^ at a high current density of 10 A g^−1^, revealing its good rate capability. In comparison, the PMTA/MWCNT-1 and PMTA/MWCNT-2 electrodes, respectively, achieve specific capacitances of ~62.8 and 128.8 F g^−1^ at 0.5 A g^−1^ and obtain only 18.3 F g^−1^ and 60 F g^−1^ when the current density increases to 10 A g^−1^. These inferior performances as compared to PMTA/MWCNT-3 can be attributed to their lower content of PMTA which accounts for abundant pseudocapacitance for the electrode. Figure 3e further displays the electrochemical impedance plots of MWCNTs, PMTA, and PMTA/MWCNT-3. The semicircles in the high frequency region indicates a charge transfer process which is controlled by the electrode reaction kinetics, while in the low frequency region, the observed slops imply the mass transfer-controlled process. The intercept of the plots on the x-axis at high frequency corresponds to the internal resistance (Rs), which mainly includes solution resistance and contact resistance. Small Rs were observed for the tested MWCNTs, PMTA, and PMTA/MWCNT-3 electrodes, implying their low internal resistances. It should be noted that faster mass diffusion was observed for the PMTA/MWCNT-3 electrode as compared to the pristine PMTA electrode, which can be explained by the assistance of MWCNTs which help alleviate the stacking and aggregation of PMTA. The cycling stability of the PMTA/MWCNT-3 electrode was also investigated, as shown in Figure 3f. The PMTA/MWCNT-3 electrode retained 89.7% of the initial capacitance after 8000 cycles at 5 A g^−1^, indicating its excellent cyclability. It should be noted that the specific capacitance (375.2 F g^−1^ at 1 A g^−1^) and cycling performance 89.7% (8000 cycles, 5 A g^−1^) of the PMTA/MWCNT electrode are competitive among the previously reported electrode materials based on porous organic polymers (Table S1).

**Figure 4:**
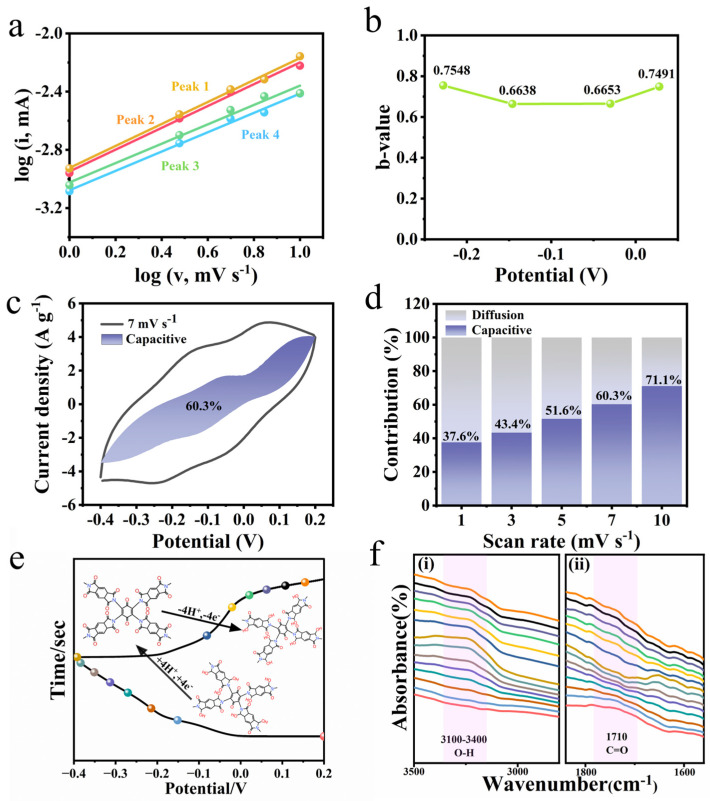
(**a**) The curve of Log (i) vs Log (v); (**b**) b values plotted against voltages; (**c**) capacitive contribution of PMTA/MWCNT-3 at a scan rate of 7 mV s^−1^; (**d**) diffusion and capacitive contributions of PMTA/MWCNT-3 at different scan rates; (**e**) reversible redox-active structural evolution, and (**f**) in situ FT-IR spectra of PMTA/MWCNTs recorded upon different charge/discharge states. The different colors of the spectra lines are corresponded to the colored dots in figure (**e**) upon different potentials.

To explore the reaction kinetics and energy storage mechanism of PMTA/MWCNTs, CV curves were collected at different sweeping rate, and the b value and capacitance contribution rate was detected and calculated according to the following Equation [36]:

(5) $$i=av^{b}$$

(6) $$i(v)=k_{1}v+kv^{1/2}$$

where i and *v* are the current and scanning rate, respectively, and a and b are adjustable parameters. k_1_ and k_2_ are constants. It is believed that the calculated b value reflects the electrochemical process during the electrode reactions. When it approaches 0.5, a diffusion-controlled behavior is assumed to exist, and if it approaches 1.0, a capacitive-controlled process is thought to occur. As exhibited in Figure 4a,b, the obtained b values are in the range 0.5–1.0, implying the co-existence of both a diffusion and a capacitive process. It worth noting that the electrochemical process changes upon scanning rates. As shown in Figure 4c,d, the capacitive contribution increases from 37.6% to 71.1% when the scanning rate increases from 1 mV s^−1^ to 10 mV s^−1^, implying a restricted diffusion process upon a high scanning rate. 

To gain more details on the electrode reactions relevant to the structure of PMTA/MWCNTs, in situ FT-IR spectra of the PMTA/MWCNT materials on the electrode were collected for detection upon different charge/discharge states, as shown in Figure 4e,f. It was observed that the intensity of the characteristic signal arising from the carbonyl groups (C=O) at around ~1710 cm^−1^ decreases upon discharge and increases upon charge. Meanwhile, the broad absorption peak in between ~3100–3500 cm⁻¹ which is attributed to the hydroxyl groups (O-H) increases upon discharge and weakens upon charge. These signal changes are related to the reversible structural evolution of between -C=O and -C-OH in PMTA/MWCNTs upon oxidation and reduction along with the removal and insertion of H+, explaining the high pseudocapacitance of PMTA/MWCNTs. This observed structural change against different potentials is in accordance with the redox peaks in CV curves involved in the electrochemical faradic reactions. It should be noted that based on the reaction mechanism, the theoretical capacity of PMTA is calculated to be ~102.3 mAh g⁻¹, while the actual capacity at 1 A g⁻¹ of PMTA/MWCNTs is measured to be ~62.5 mAh g⁻¹. This observation demonstrates that even with the assistance of MWCNTs, there is still large room for the improvement of the material’s performance.

**Figure 5:**
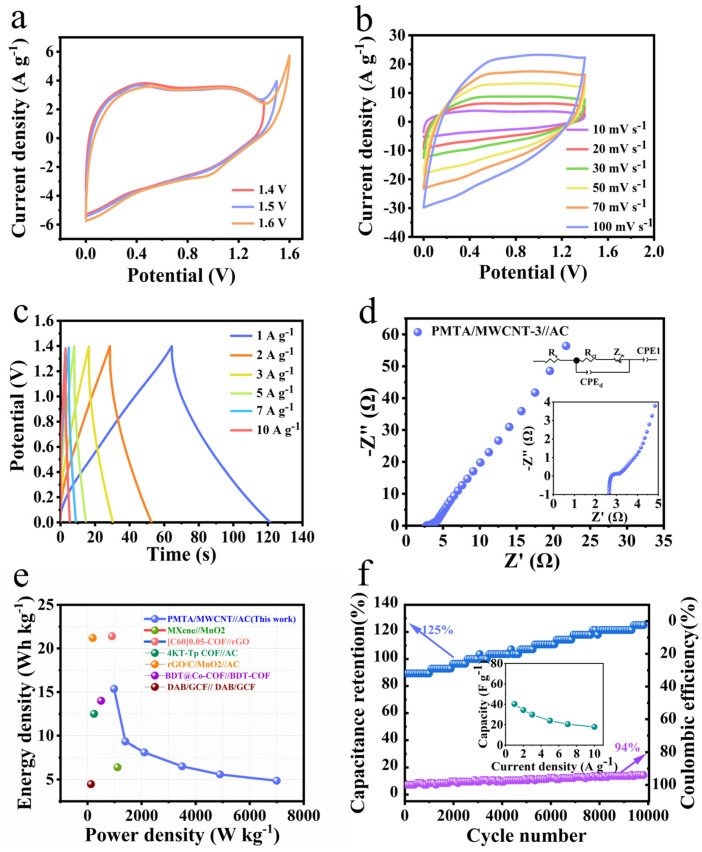
Electrochemical performance of the PMTA/MWCNT-3//AC ASC device: (**a**) CV curves at different potential windows at 10 mV s^−1^; (**b**) CV curves collected at different sweeping rates; (**c**) GCD curves obtained at different current densities; (**d**) Nyquist plots (inset: region of high frequency); (**e**) Ragone plots of the PMTA/MWCNT-3 ASC device and previously reported ASCs; (**f**) cycling capability and Coulombic efficiency of the ASC at 5 A g^−1^ (inset: the specific capacity of the ASC at distinct current densities).

To evaluate the practical potential of PMTA/MWCNTs in supercapacitors, we assembled an asymmetric supercapacitor (ASC), PMTA/MWCNT-3//AC, using 1M H_2_SO_4_ as the electrolyte, PMTA/MWCNT-3 as the positive electrode, and AC as the negative electrode. The CV curves of PMTA/MWCNT-3//AC tested with different voltage windows indicate an obvious polarization phenomenon at 0–1.6 V (Figure 5a). Therefore, a working window of 0–1.5 V was adopted for the ASC. The similar shapes for the CV curves of the PMTA/MWCNT-3//AC device at different scanning rates (i.e., 10 to 100 mV s^−1^) indicate the excellent reversibility and structural stability of the electrode materials in the ASC device (Figure 5b) [37]. Rate capability is also one of the important parameters that cannot be ignored in the practical application of supercapacitors. Figure 5c shows the GCD curves of the ASC device at different current densities. The specific capacitances are calculated to be 46, 45, 41, 39, 37, and 28 F g^−1^ at 1, 2, 3, 5, 7, and 10 A g^−1^, respectively. The assembled ASC device still maintained a retention rate of 61% when current density increased from 1 to 10 A g^−1^, indicating its good rate performance. Figure 5d shows a small intercept on the x-axis which can be directed to the Rs (2.7 Ω) and implies a low internal resistance for the ASC. Figure 5e shows the Ragone diagram of the ASC device. The assembled PMTA/MWCNT-3//AC ASC shows an energy density of 15.4 Wh kg^−1^ when the power density is 980.4W kg^−1^. In addition, a long-term cycling performance is observed for the assembled ASC, i.e., a high retention rate of 125% and a Coulombic efficiency (CE) of 94% were observed after 10,000 cycles at 5 A g^−1^ (Figure 5f), proving its excellent cyclability and further indicating the great potential of the assembled PMTA/MWCNT-3//AC device for practical application. It is worth noting that the electrochemical performances of the PMTA/MWCNT-3//AC device are competitive to the previously reported POP-based ASC devices (Table S2) [26,38,39,40,41,42].

## 4. Conclusions

In summary, a redox-active polyimide-based POP PMTA is prepared in situ with MWCNTs using a one-step solvothermal method. Thanks to its rich mesoporous structure, improved conductivity, and enhanced utilization of carbonyl groups achieved with the assistance of MWCNTs, the PMTA/MWCNT electrode exhibits good electrochemical performances. In the three-electrode system, it achieves a high specific capacitance of 398 F g^−1^ at 0.5 A g^−1^ and an excellent cycling stability of 89.7% after 8000 cycles at 5 A g^−1^. The assembled PMTA/MWCNT-3//AC ASC device exhibits a maximum energy density of 15.4 Wh kg^−1^ at 980.4 W kg^−1^ and excellent cycling performance, with a capacity retention rate of 125%, and a CE of 94% after 10,000 cycles at 5 A g^−1^. Electrochemical reaction kinetics and in situ FT-IR are studied in detail to reveal the energy storage mechanism of these electrodes.

## Data Availability

The data presented in this study are available on request from the corresponding author.

## Supplementary Materials

The following supporting information can be downloaded at: https://www.mdpi.com/article/10.3390/nano14171388/s1, Figure S1: (a) SEM of PMTA; (b) TEM of PMTA; Figure S2: EDS (a) spectrum and (b) mapping of PMTA/MWCNTs showing C, N and O elements; Figure S3: Nitrogen adsorption/desorption isotherms of PMTA. Insert: pore size and distribution of PMTA.; Figure S4: (a) CV curves and (b) GCD curves of PMTA/MWCNT-1, at a scan rate of 5, 10, 20, 30, and 50 mV s^−1^ and a current density of 0.5, 1, 2, 3, 5, 7, and 10 A g^−1^, respectively; (c) CV curves and (d) GCD curves of PMTA/MWCNT-2, at a scan rate of 5, 10, 20, 30, and 50 mV s^−1^ and a current density of 0.5, 1, 2, 3, 5, 7, and 10 A g^−1^, respectively; Table S1: Comparison of electrochemical properties of porous organic polymers in a three-electrode system; Table S2: Summary of electrochemical performance of different assembled asymmetric supercapacitors.
