# Supplementary material for: Boosting of Redox-Active Polyimide Porous Organic Polymers with Multi-Walled Carbon Nanotubes towards Pseudocapacitive Energy Storage
Source: Nanomaterials (Basel). 2024 Aug 26;14(17):1388. doi: 10.3390/nano14171388 (PMC11397463; doi:10.3390/nano14171388)
Supplement: Supplementary file 1 [file nanomaterials-14-01388-s001.zip › nanomaterials-3154960-supplementary.docx]

**Supporting Information**

**Boosting of Redox Active Polyimide Porous Organic Polymers with Multi-walled Carbon Nanotubes towards Pseudocapacitive Energy Storage**

Tian Zhou^†1^, Yu Yuan^†1^, Luyi Xiao^1^, Wei Ding, Yong Wang^1,2^, Li-Ping Lv^1,2^*

*^1^School of Environmental and Chemical Engineering*

*^2^Key Laboratory of Organic Compound Pollution Control Engineering (MOE)*

*Shanghai University, 99 Shangda Road, Shanghai, P. R. China, 200444*

*Corresponding authors: Tel: +86-21-66137723

Email address: [liping_lv@shu.edu.cn](mailto:liping_lv@shu.edu.cn)


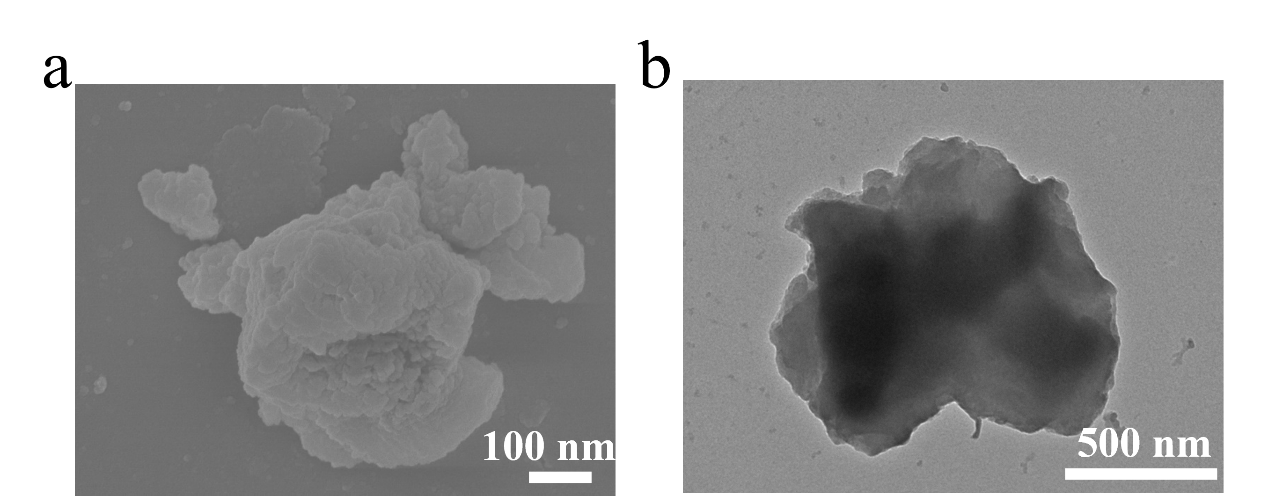


Figure S1. (a) SEM of PMTA; (b) TEM of PMTA.


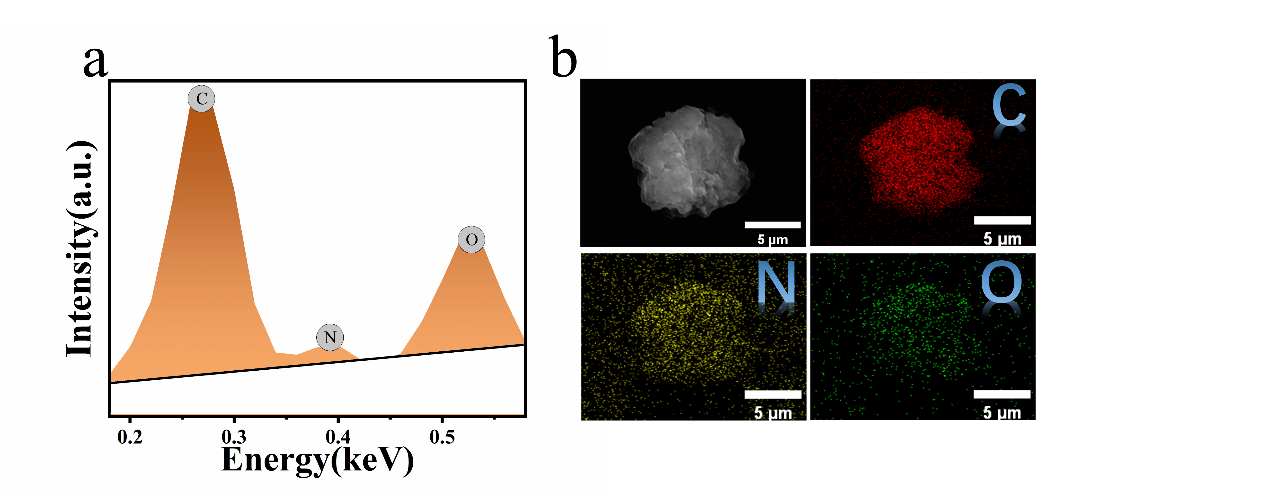


Figure S2. EDS (a) spectrum and (b) mapping of PMTA/MWCNTs showing C, N, and O elements.


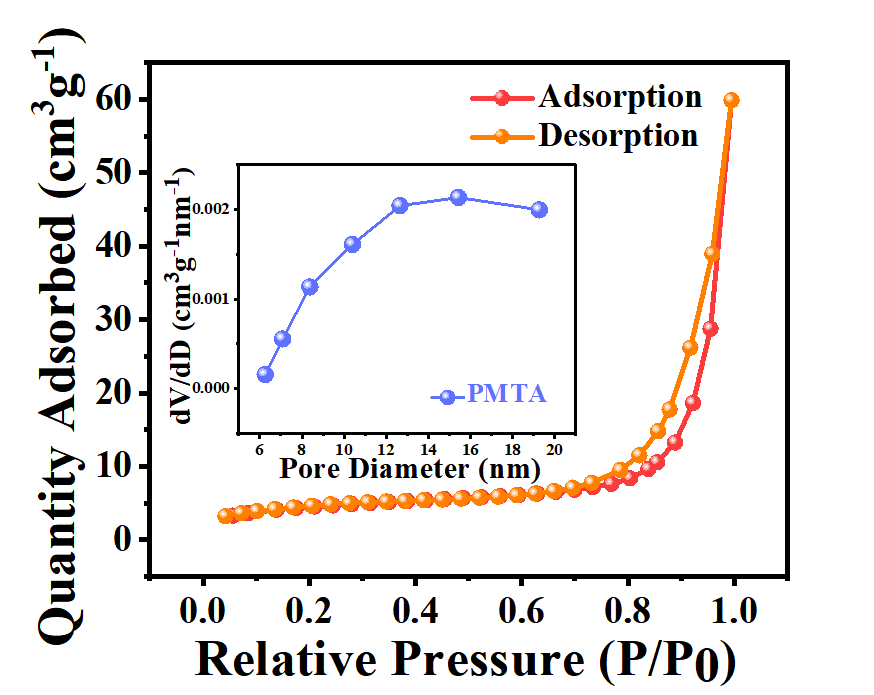


Figure S3. Nitrogen adsorption/desorption isotherms of PMTA. Insert: pore size and distribution of PMTA.


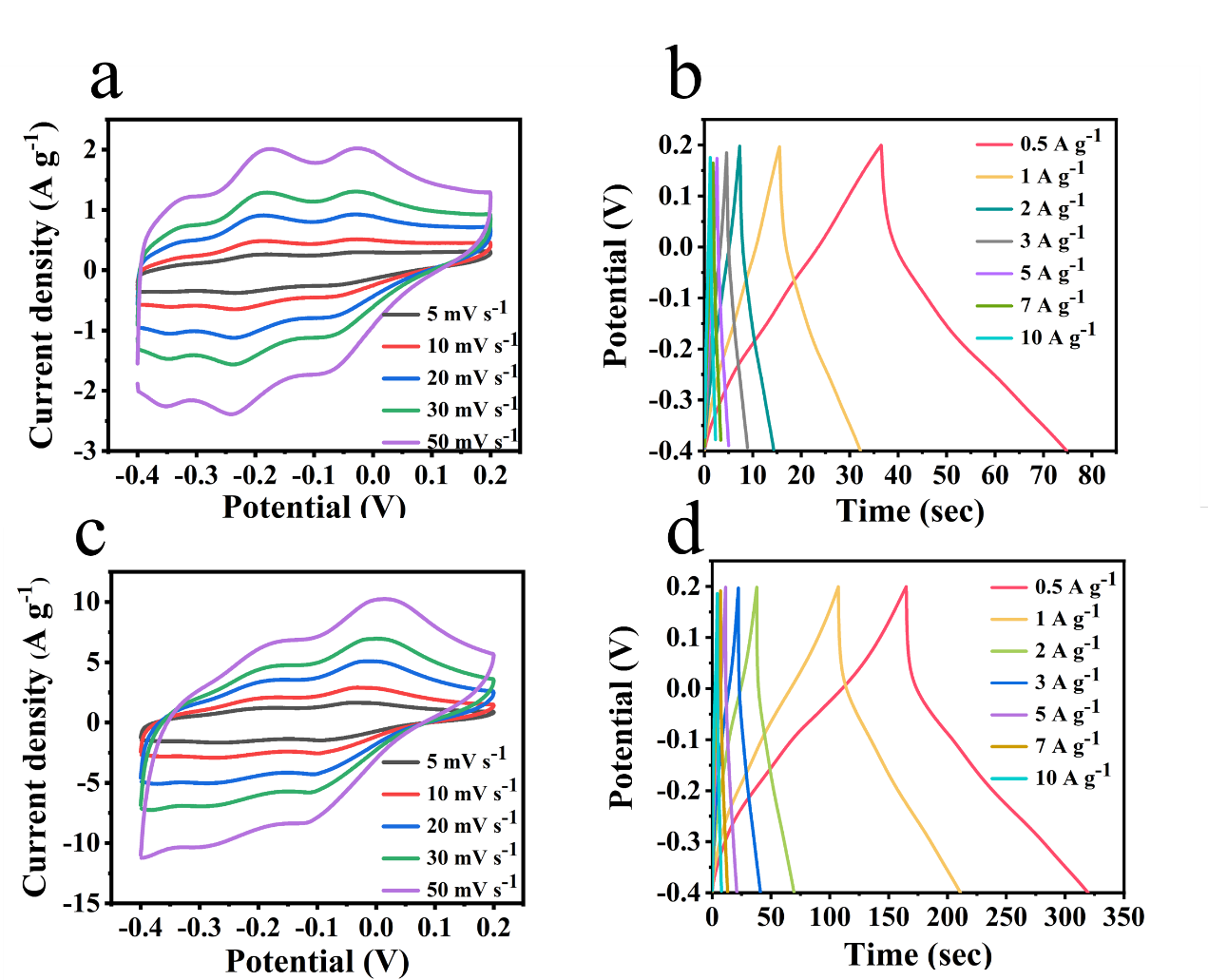


Figure S4. (a) CV curves and (b) GCD curves of PMTA/MWCNT-1 at a scan rate of 5, 10, 20, 30, and 50 mV s^−1^ and a current density of 0.5, 1, 2, 3, 5, 7, and 10 A g^−1^, respectively; (c) CV curves and (d) GCD curves of PMTA/MWCNT-2 at a scan rate of 5, 10, 20, 30, and 50 mV s^−1^ and a current density of 0.5, 1, 2, 3, 5, 7, and 10 A g^−1^, respectively.

Table S1. Comparison of electrochemical properties of porous organic polymers in a three-electrode system.

| **Materials** | **Specific capacitance,**  **current density** | **Capacity retention,**  **cycle number** | **Ref.** |
| --- | --- | --- | --- |
| **PMTA/MWCNT** | **375.2 F g^–1^ at 1 A g^–1^** | **89.7% (8000, 5 A g^–1^)** | **This work** |
| **DAAQ-COFs/GA** | **378 F g^–1^ at 1 A g^–1^** | **87.8% (20000, 15 A g^–1^)** | **J. Mater. Chem. A,2021, 9, 16824** |
| **SWCNTs-TpPa-COFs** | **153F g^–1^ at 0.5 A g^–1^** | **114% (2000, 2 A g^–1^)** | **Chin. Chem. Lett. 28 (2017) 2269–2273** |
| **Tp-AT-POP** | **348.3 F g^–1^ at 0.5 A g^–1^** | **71% (5000, 5 A g^–1^)** | **J. Ind. Eng. Chem. 123 (2023) 320–329** |
| **CMP-800** | **375F g^–1^ at 0.5 A g^–1^** | **98.8% (1000, 5 A g^–1^)** | **J. Energy Storage 32 (2020) 101786** |
| **PDC−MA− COF** | **331F g^–1^ at 1 A g^–1^** | **78% (9000, 5 A g^–1^)** | **ACS Appl. Mater. Interfaces 2019, 11, 26355−26363** |
| **N-PC** | **112F g^–1^ at 1 A g^–1^** | **89.4% (5000, 2 A g^–1^)** | **Carbon Lett. (2021) 31:879–886** |
| **NIBDZ** | **88.4F g^–1^ at 0.5 A g^–1^** | **93.6% (5000, 0.5 A g^–1^)** | **Eur. Polym. J.** (**2017)** **0014-3057** |
| **TTT-DHTD** | **273 F g ^–1^ at 0.5 A g^–1^** | **51% (2000, 1 A g^–1^)** | **ACS Appl. Energy Mater. 2023, 6, 9256−9263** |
| **CNT@TFA-COF** | **338 F g^–1^ at 1 A g^–1^** | **86% (7000, 5 A g^–1^)** | **Dalton Trans., 2023, 52, 2762** |

Table S2. Summary of electrochemical performance of different assembled asymmetric supercapacitors.

| **Device** | **Energy density (W h kg^–1^), power density (W kg^–1^)** | **Capacity retention, cycle number** | **Ref.** |
| --- | --- | --- | --- |
| **PMTA/MWCNT//AC** | **15.4, 980.4** | **125% (10000, 5 A g^–1^)** | **This work** |
| **CMFs//CCMP** | **19.7, 7000** | **88.9% (20000, 5 A g^–1^)** | **Chem. Eng. J. 458 (2023) 141434** |
| **[C_60_]0.05-COF//rGO** | **21.4, 900** | **99% (5000, 4 A g^–1^)** | **Carbon 182 (2021) 144e154** |
| **4KT-Tp COF//AC** | **12.5, 240** | **99% (10000, 5 A g^–1^)** | **CCS Chem. 2020, 2, 696–706** |
| **rGO@COFs-Ni_12_P_5_// AC** | **18.75, 7500** | **84.6% (5000, 10 A g^–1^)** | **J. Energy Storage 97 (2024) 112616** |
| **BDT@Co-COF//BDT-COF** | **14, 500** | **75% (5000, 5 A g^–1^)** | **J. Alloys Compd. 970 (2024) 172634** |
| **DAB/GCF// DAB/GCF** | **4.47, 125** | **100% (20000, 10 A g^–1^)** | **Chem. Eng. J. 449 (2022) 137858** |
